# Digital, Media, and Information Literacies and the Well-Being of Neurotypical and Neurodivergent Children: A Synthetic Knowledge Synthesis

**DOI:** 10.3390/healthcare14162645

**Published:** 2026-08-20

**Authors:** Irena Lovrenčič Držanič, Suzana Žilič Fišer, Laura Horvat, Helena Blažun Vošner, Peter Kokol

**Affiliations:** 1 Faculty of Electrical Engineering and Computer Science, University of Maribor, 2000 Maribor, Slovenia; suzana.zilicfiser@um.si (S.Ž.F.); laura.horvat3@um.si (L.H.); peter.kokol@um.si (P.K.); 2 Community Healthcare Center Dr. Adolf Drolc Maribor, 2000 Maribor, Slovenia; helena.blazun@zd-mb.si

**Keywords:** digital literacies, neurodiversity, child well-being, digital resilience, inclusive pedagogy, cyberbullying, children, synthetic knowledge synthesis

## Abstract

**Background/Objectives:** Children now spend a substantial part of daily life in digital environments, and their digital, media, and information literacies shape their online safety, social-emotional development, and mental health, making these competencies a concern for child public health and preventive paediatric care. This study applies a Synthetic Knowledge Synthesis (SKS) to map how digital, media, and information literacies (hereafter digital literacies) among children have evolved, comparing neurotypical and neurodivergent children. SKS is a semi-automated approach that combines descriptive bibliometrics, keyword co-occurrence mapping, and qualitative content analysis to map an entire research field or topic. **Methods:** We treat digital literacies as relevant to children’s health, well-being, and safe online participation, and we analyse Scopus-indexed literature from 1996 to 2025 using descriptive bibliometrics, keyword co-occurrence mapping, and qualitative content analysis. **Results:** The mapping shows strong growth in general digital-literacy scholarship alongside a small but persistent body of work on a “double digital divide” affecting children with autism spectrum disorder (ASD), ADHD, and dyslexia. The two bodies of work differ in emphasis: the general literature foregrounds social integration and critical agency, while research on neurodivergent children foregrounds inclusive pedagogy, implicit learning, and multimodal expression. It also shifts from general digital-safety awareness toward protective mediations for vulnerabilities such as social-emotional decoding differences and impulsivity, which bear on children’s online safety and mental health. Because bibliometric mapping reveals where research is concentrated rather than what works in practice, the synthesis points to evidence gaps rather than proven methods. **Conclusions:** On this basis, we argue for an integrated public-health and socio-educational framework that complements universal literacy standards with adaptive, assistive safety nets, so that all children can take part in digital life safely and on equal terms.

## 1. Introduction

In the contemporary digital landscape, the pervasiveness of screen time and interconnected platforms has been associated with youth mental health, with a growing body of research linking heavy or poorly regulated digital exposure to paediatric anxiety, depressive symptoms, and sleep disruption [1,2,3], although the strength and direction of these associations remain debated. Consequently, the ability to navigate, evaluate, and create media has transitioned from a specialised skill set to a fundamental prerequisite for social, civic, and educational participation [4,5,6]. The convergence of information, media, and digital literacies (hereafter digital literacies) forms a multi-dimensional framework essential for the development of modern youth, who are increasingly required to manage complex information environments [6,7]. While early “digital native” discourses suggested a natural, inherent proficiency among children, subsequent research has demonstrated that digital competencies are not evenly distributed and require structured pedagogical support to ensure equity [8,9,10]. For children, these literacies are deeply embedded in their participatory culture and are central to developing a sense of agency in a networked world [11,12]. For paediatric and developmental research, these competencies also shape children’s online safety, social-emotional well-being, and mental health, positioning digital literacies as a legitimate concern for child-health scholarship and practice.

Despite the rapid expansion of this field, a significant portion of the academic literature has historically focused on neurotypical development, often overlooking the unique digital experiences and requirements of neurodivergent populations [13,14]. Children with autism spectrum disorder (ASD), attention-deficit/hyperactivity disorder (ADHD), and other neurodevelopmental conditions often exhibit distinct patterns of technology use, ranging from heightened engagement with specific digital platforms to unique challenges in online social navigation and executive function [15,16]. While technology offers significant potential as a compensatory tool for communication and learning [17,18], neurodivergent children may also face specific vulnerabilities, such as increased risks related to digital addiction [19,20] or social isolation [21].

While current educational frameworks have established a socio-educational context for digital literacies [22], these models often assume a neurotypical learner [22]. This creates a “double digital divide” for children with special educational needs, such as autism, ADHD, and dyslexia [23], which represents a health-disparity and health-equity problem. In these populations, traditional instructional strategies often fail to account for specific cognitive barriers in literacy acquisition, such as difficulties with explicit grammar or social-emotional decoding [24]. While digital tools like gamification and digital storytelling offer promising “assistive” pathways, there is little integrated research on how rapport-building and specialised digital parenting can mitigate the heightened risks of cyberbullying and exclusion for neurodivergent children [25,26,27]. Viewed in health terms, this divide is also a child-health-equity problem, because the children least served by mainstream digital-literacy provision are frequently those most exposed to online harms that affect mental health, such as cyberbullying, victimisation, and social isolation. Building digital literacy in these cohorts is therefore not only a pedagogical goal but may also help cultivate digital resilience, which has been proposed as a psychosocial protective factor against online harm.

Understanding the academic trajectory of this field is important for identifying research gaps and regional disparities in digital literacy support. Synthetic knowledge synthesis (SKS) [28,29] provides a rigorous qualitative and quantitative lens to map the evolution of these concepts, tracking the transition from broad literacy definitions to more inclusive learning environments and frameworks, also taking into account also neurodiversity-focused paradigms. SKS is a semi-automated approach that combines descriptive bibliometrics, keyword co-occurrence mapping, and qualitative content analysis, and it analyses the complete set of research publication metadata harvested by a search rather than a selected set of publications [28,29]. This study aims to map how digital literacies among children have evolved, comparing neurotypical and neurodivergent children. By examining publication trends, geographic distributions, and the qualitative outputs of the synthesis, it highlights the growing but still emerging scholarly focus on narrowing the gap between digital-literacy frameworks and the specific needs of both groups. For child-health and public-health audiences, locating where this evidence is concentrated, and where it is missing, is a prerequisite for deciding where to direct preventive and protective resources for children online. More precisely, the following research questions have been posed:

- What are the longitudinal quantitative and geospatial trends within the existing scholarship regarding children’s digital literacies?
- How does the literature frame educational and parental mediation as a means of bridging digital-literacy divides among children?
- How does the literature address the integration of inclusive pedagogical approaches in supporting digital-literacy acquisition and socio-emotional resilience, particularly for neurodivergent children?
- Which digital-parenting strategies and protective mediations does the literature emphasise in relation to cyberbullying and online victimisation among children, and where do the principal evidence gaps lie?

The search strings define the field, not the individual research questions. The concepts named in RQ2 to RQ4 are not search terms; they emerged as keyword groupings within the retrieved corpora and are answered through the thematic clusters and their representative publications, which are reported in Section 3.2, Section 3.3 and Section 3.4.

## 2. Materials and Methods

Synthetic Knowledge Synthesis is a semi-automated framework for reviewing a body of research. It begins by extracting bibliographic records and metadata from Scopus, chosen for its broad coverage, including non-English work, and its detailed metadata. SKS then combines three analyses: descriptive bibliometrics to quantify publication trends and leading contributors; keyword co-occurrence mapping in VOSviewer v1.6.21 to identify thematic clusters; and qualitative content analysis to interpret the main findings and emerging topics. Working with whole corpora in this way reduces selection bias and supports a reproducible overview of a field and its likely directions. We applied SKS independently to two corpora, the All Children Corpus (ACC) and the Neurodivergent Children Corpus (NCC), and then compared the results. In the text which follows, general and all children refer to the ACC, which covers research on children as a whole, while neurodivergent refers to the NCC; because the NCC query is a restriction of the ACC query, the NCC records are contained in the ACC, so general should not be read as strictly neurotypical. The historical analysis used the bibliographic time-slicing approach [30]: the corpora were divided into consecutive time periods, and publication activity and thematic emphasis were examined across these slices to trace how the field has developed. Because this synthesis concerns the bearing of digital literacies on children’s online safety, social-emotional development, and mental health, Scopus also suited the topic, given its broad indexing of biomedical, public-health, and behavioural-science research alongside education and media studies. By avoiding the restrictive exclusion criteria of traditional systematic reviews, SKS is well suited to mapping this emerging public-health literature.

The corpora were harvested on 15 March 2026 using the following two search strings:

TITLE-ABS-KEY((“media literacy” OR “digital literacy” OR “information literacy”) AND child*) for ACC.

TITLE-ABS-KEY((“digital literacy” OR “media literacy” OR “information literacy”) AND child* AND (autism OR ADHD OR dyslexia OR neurodiv*)) for NCC.

Corpus scope and completeness. Each corpus was analysed in its entirety: the complete set of records returned by the two Scopus queries above was retained, and no eligibility screening or manual exclusion of individual records was performed. Because the records were drawn from a single database, no cross-database de-duplication was required. Beyond the terms of the search strings, no document-type or language restrictions were applied; the retrieved records span 1996–2025. Analysing whole corpora rather than a screened subset is a deliberate feature of SKS that, together with the use of a single, consistently indexed database, supports a reproducible overview of the field as represented in Scopus. The population of interest, children, was operationalised through the child* operator in the search strings; no additional age restriction was applied, since SKS maps a research field rather than individual interventions (see Section 5 for the corresponding limitation). For the NCC, neurodevelopmental profiles were captured with the terms autism OR ADHD OR dyslexia OR neurodiv*, so children with other neurodevelopmental profiles may not be represented. Scopus supplies titles, abstracts, and keywords in English also for work published in other languages, and we translated no records, so back-translation and verification of translated terminology did not arise (see Section 5). Because there is no screening stage, PRISMA and comparable reporting frameworks do not apply; reproducibility rests on the search strings, the harvest date, and the mapping parameters reported here.

Bibliometric mapping parameters. Thematic mapping was performed in VOSviewer using keyword co-occurrence analysis, with the association strength normalisation (the VOSviewer default). The unit of analysis was an author keyword, and the counting method was full counting. For the ACC, the minimum occurrence threshold was set to 17, which selected 76 terms following Zipf’s law; the layout parameters were attraction 3, repulsion 0, and resolution 1.4. For the NCC, the minimum occurrence threshold was set to 1 because of the small corpus; all terms unrelated to digital literacies and children’s neurodivergence were then removed manually, leaving 34 terms on the map. The layout parameters for the NCC were attraction 4, repulsion 0, and resolution 1. No thesaurus file was used, so no synonyms or spelling variants were merged; the terms shown on the NCC map were selected manually in the VOSviewer interface. Variant forms of the same concept therefore appear as separate nodes. This manual curation operated at the level of the map, not the corpus: the complete set of 32 NCC records was analysed, while the set of keywords displayed for the NCC was curated manually because the corpus is small and noisy. The resulting maps comprised five thematic clusters for the ACC and three for the NCC, distinguished by colour; the cluster colours, labels, and representative keywords reported in Section 3 derive from this output.

Qualitative content analysis. The clusters generated by VOSviewer were interpreted and assigned thematic labels through qualitative content analysis. The unit of analysis for this qualitative step was the set of dominant keywords and their network associations in each cluster together with representative publications, not the full texts of all records. For each cluster, we read the dominant keywords and the representative publications and assigned a thematic label on that basis. For example, the green ACC cluster combines keywords on family literacy, primary education, technology, and digital literacies, which led to the label Co-learning of digital literacies. The labelling was carried out by one author (P.K.), so no inter-coder agreement was calculated, and no consensus procedure applies. To keep the step auditable, the keywords and representative publications underlying each label are reported in Section 3. These labels are interpretative and authorial: they are a standard part of bibliometric synthesis, and other analysts might group or name the terms differently, particularly for the small NCC (see Section 5).

## 3. Results

The search resulted in 2433 publications in the ACC and 32 publications in the NCC. Document types were not restricted, so the corpora include all Scopus-indexed document types returned by the queries. The SKS analysis examined the body of scholarship on digital literacies in children, with a specific focus on the intersection with neurodivergent populations (including autism, ADHD, and dyslexia). By synthesising data from Scopus, the authors evaluated the temporal evolution of this field from 1996 to 2025, as well as the geographic distribution of research contributions. Given the small size of the NCC (*n* = 32), the bibliometric mapping and clustering reported for this corpus should be read as exploratory and qualitative rather than as statistically representative; it is used to characterise the orientation of an emerging literature, not to quantify it.

### 3.1. Temporal Evolution and Growth Trends (RQ1)

The broader field of literacy research among children has demonstrated significant growth over the last three decades. Initial scholarly activity recorded in 1996 was minimal, but the field experienced a notable acceleration starting around 2015. In the general literacy domain, publication counts rose from 87 in 2015 to 260 in 2024 and 390 in 2025, exhibiting a pattern of sustained and accelerating growth.

**Figure 1:**
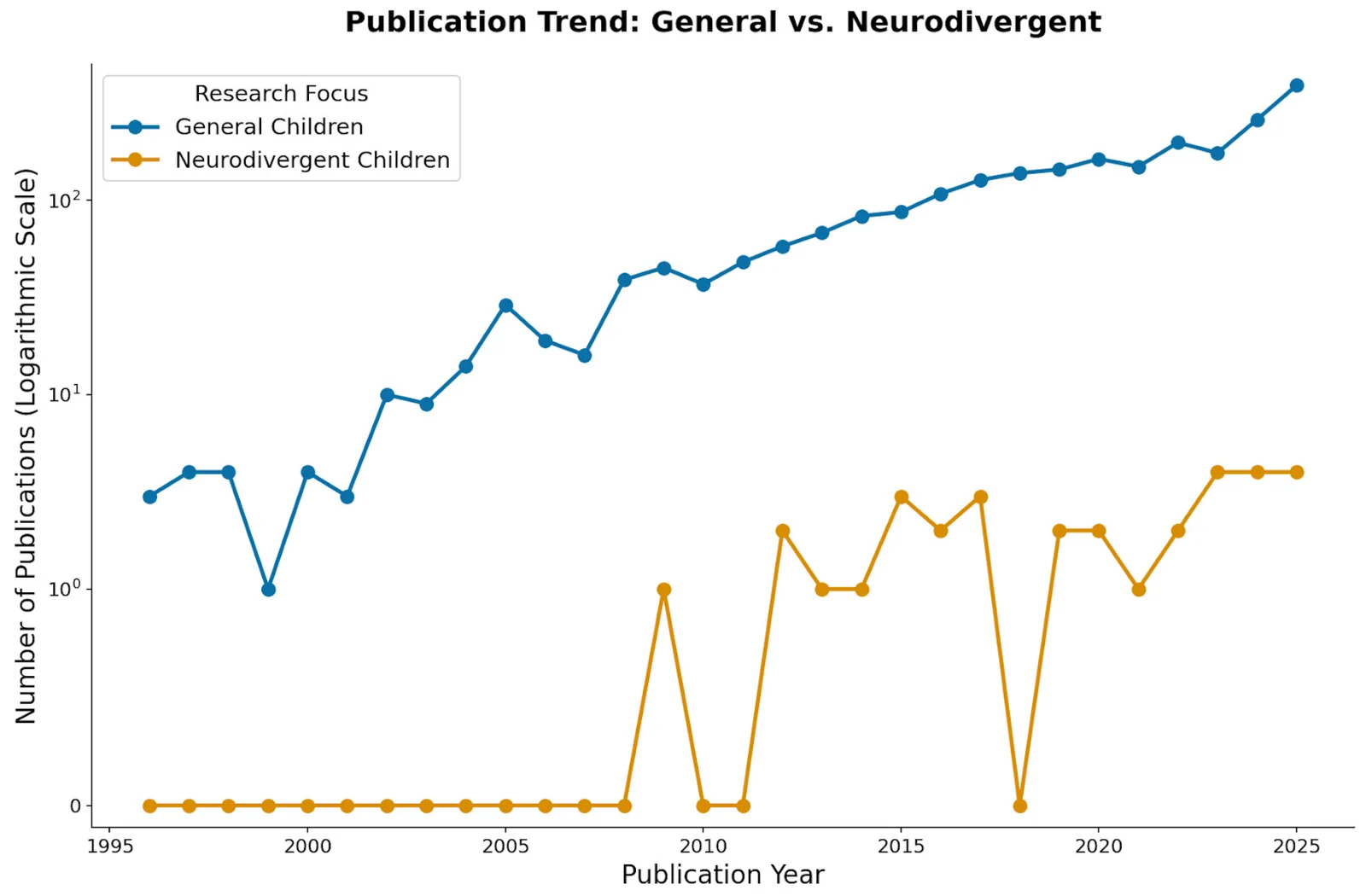
Evolution of research publications (1996–2025).

In contrast, the specialised intersection of literacy and neurodiversity is a newer subfield. While the volume is significantly smaller, averaging 4 publications annually in recent years, there is a stable presence of research, suggesting a specialised and persistent niche within the broader discourse (see Figure 1).

The geographic distribution of this research shows a high concentration in Western nations, though it maintains a global reach across 111 identified countries or territories. Because Scopus attributes a record to each contributing country, the country counts sum to 2901 and exceed the number of records.

**Figure 2:**
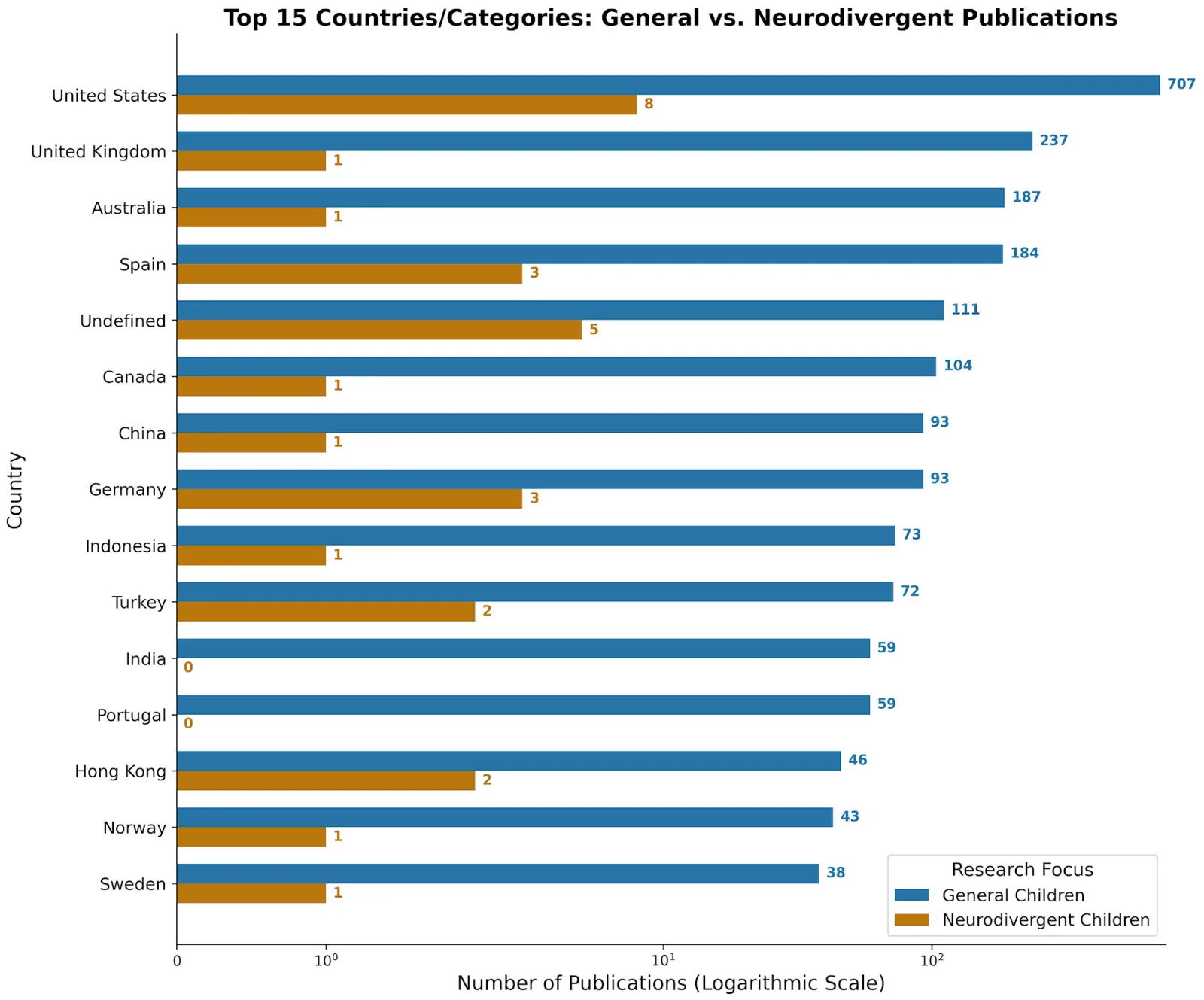
Geographical distribution: top 15 countries (general vs. neurodiversity focus). The blue bars represent total publications in the general field, while the red diamonds indicate the specific contribution to the neurodiversity subset for each country.

- Global Leaders: The United States occupies a dominant position in both the general and specialised datasets, contributing 707 publications to the general field and 8 to the neurodiversity subset. The United Kingdom (237) and Australia (187) follow as major contributors to general literacy research.
- Specialised Contributions: As shown in Figure 2, countries such as Germany and Spain show a higher proportional commitment to neurodiversity-related literacy research relative to their total output in the general field. Other contributing nations include Turkey, Hong Kong, and Italy, indicating an international interest in the digital inclusion of neurodivergent learners. These proportions rest on small numerators and indicate presence or absence of attention rather than measuring national research priorities.

The bibliometric data show an expanding body of research on digital literacies. While the general literature on children is well-established and growing exponentially, the specific application of these frameworks to neurodivergent populations is a developing area of study. The findings suggest a need for increased cross-disciplinary research to bridge the gap between general literacy models and the specific needs of neurodivergent learners.

### 3.2. Thematic Analysis

#### 3.2.1. All Children (ACC) (RQ2, RQ3)

**Figure 3:**
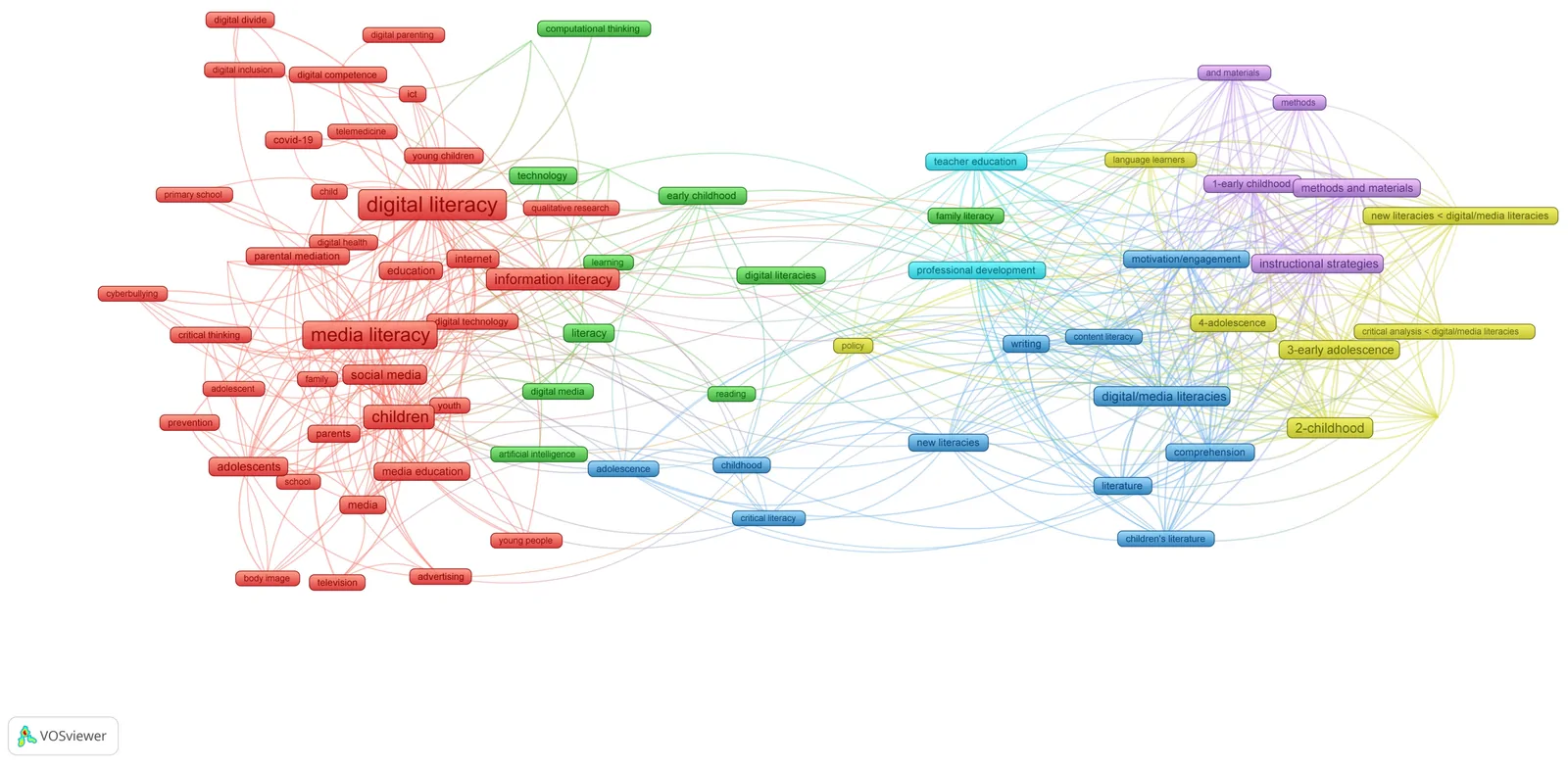
The research landscape of digital literacies–ACC. Colours denote: red, the Socio-Educational Landscape of Digital, Information and Media Literacies; green, Co-learning of Digital Literacies; blue, Applied Linguistics and New Literacy Studies; yellow, Digital Empowerment in Childhood; violet, Applied Media Pedagogy.

SKS revealed that digital literacy research can be divided into five distinct thematic clusters (Figure 3 and Table 1). These clusters show how digital literacies intersect with education, family dynamics, and emerging technologies.

The Red Cluster: The Socio-Educational Landscape of Digital, Information, and Media Literacies. This is the broadest cluster, focusing on the societal and ethical dimensions of literacy [31,32,33,34]. It looks at the digital divide [35,36] and digital inclusion [33,36], ensuring that literacy is not just a skill but a means of social equity [34,37,38].

- Key Actors: Primary school children, parents, and the broader community.
- Critical Issues: It uniquely addresses the risks and responsibilities of the digital world, such as cyberbullying, parental mediation, and the development of critical thinking to navigate social media.

The Green Cluster: Co-learning of digital literacies. This cluster emphasises the collaborative nature of learning [39,40,41,42,43], particularly within the family unit (family literacy). It bridges the gap between home and primary education.

- Key Actors: Families and early primary students.
- Technological Frontier: This cluster is the only one to mention Artificial Intelligence (AI), suggesting a focus on how children and families adapt to the newest waves of automated technology.

The Blue Cluster: Applied Linguistics and New Literacy Studies. This cluster is more theoretical and professional. It moves away from the “what” of literacy and looks at the “how” through the lens of linguistics [41,44,45]. It focuses on the cognitive aspects of learning, such as comprehension and motivation.

- Key Actors: Teachers and educators.
- Professional Development: A major pillar here is teacher education, ensuring that those instructing children have the critical and content literacy necessary to teach in a digital age.

The Yellow Cluster: Digital empowerment in childhood. This cluster targets the semiotic and visual aspects of the digital world [46,47]. As video and icons [48] increasingly dominate online media, visual literacy [49] is treated as a tool for empowerment [50,51].

- Key Actors: Children as active analysts.
- Approach: It encourages critical analysis of digital media, moving the child from a passive consumer to an empowered, critical viewer who understands how digital messages are constructed.

The Violet Cluster: Applied Media Pedagogy. This is the most specialised and practical cluster. It focuses specifically on instructional strategies [52,53,54,55].

- Key Actors: Early childhood educators and learners.
- Context: While other clusters look at primary school or general childhood, this one zooms in on early childhood, seeking the specific teaching methods required for the youngest learners to begin their media journey.

SKS ends with themes: categories are formed from the links, and proximity between keywords within each cluster, and the categories are then condensed into themes [28]. The macro, meso and micro levels below are not a further step of the method but our reading of the five themes once they had been established. We ordered them by the scope of the actors and the setting each theme addresses. A theme was assigned to the macro level when its keywords concern society as a whole and the conditions of participation in it, to the meso level when they concern the settings in which children learn, that is, families, schools and teacher training, and to the micro level when they concern individual skills or the methods used to teach them. The assignment follows directly from the representative keywords: society-wide terms such as digital divide, digital inclusion and cyberbullying place the red theme at the macro level; family literacy, primary education and teacher education place the green and blue themes at the meso level; and visual literacy, content literacy and instructional strategies place the yellow and violet themes at the micro level. The levels describe the scope of the research attention within each theme, not a hierarchy of importance or of evidential strength:

- Macro (Red): Society, inclusion, and digital safety.
- Meso (Green & Blue): The systems of learning: families, schools, and teacher training.
- Micro (Yellow & Violet): Specific skills (visual literacy, content literacy) and specific classroom methods (instructional strategies) for the youngest learners.

Together, they form a holistic view of digital literacies that encompasses social justice, cognitive development, family collaboration, and classroom practice.

#### 3.2.2. Neurodivergent Children (RQ3, RQ4)

**Figure 4:**
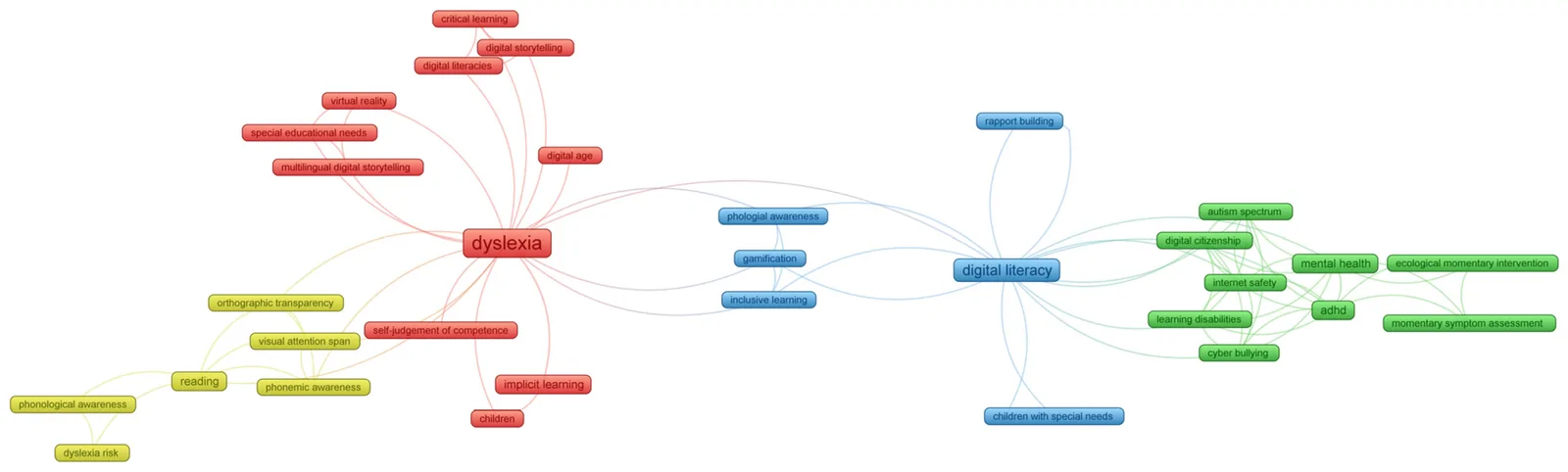
The research landscape of digital literacies–NCC. Colours denote: red and yellow, Inclusive Digital Pedagogy and Implicit Language Acquisition in Neurodiverse Learners; blue, Relational and Gamified Frameworks for Inclusive Digital Literacy; green, Digital Risk and Protective Mediations for Neurodivergent Learners.

The thematic analysis for neurodivergent children is based on the research landscape presented in Figure 4, and the themes and categories are shown in Table 2. The themes focus specifically on Neurodiversity, Special Educational Needs (SEN), and Inclusive Digital Practices. While the first research landscape is focused on general education and society, this second one is formed from three distinct, specialised clusters.

The first theme, Inclusive Digital Pedagogy and Implicit Language Acquisition in Neurodiverse Learners, is focused on the “internal” mechanics of how children with learning differences process language. It moves away from rigid, rule-based teaching and toward implicit learning, allowing dyslexic children to absorb grammar and spelling naturally through digital interaction [56]. The key tool is digital storytelling [57]. This is used as a “critical literacy” bridge, allowing children who may struggle with traditional writing to express complex ideas using images, sound, and sequence.

The theme Relational and Gamified Frameworks for Inclusive Digital Literacy emphasises that technology alone does not produce learning: the emotional and relational context matters as much as the tools themselves. It treats rapport building as both a clinical and a pedagogical requirement, and centres on digital parenting and gamification [58,59]. The parenting strand examines how parents of children with special needs accompany them online and help them manage digital environments [60]. Gamification uses game-based mechanics to lower anxiety and raise engagement, so that learning to use digital tools feels rewarding rather than frustrating. In both cases, the aim is a supportive social-emotional setting for digital development [61].

The theme Digital Risk and Protective Mediations for Neurodivergent Learners addresses the more challenging side of the digital world, with attention to autism and ADHD profiles [62]. It notes that these children may face higher risks due to impulsivity or difficulties in reading social cues online [63]. Key issues are cyberbullying and internet safety [64,65]. The research here seeks protective mediations, strategies that protect these children without excluding them from the benefits of the internet, aiming to build digital resilience and specialised safety nets for the most vulnerable online users [66,67].

Applying the same criterion as in Section 3.2.1, the three themes of the NCC sit at the meso and micro levels: the settings of learning and the relationship between child, parent and educator, and the individual cognitive profile. No theme occupies the macro level, whereas in the ACC the broadest theme does. Given the size of the NCC and the manual curation of its term map, this should be read as an indication of where research attention has so far been directed, not as evidence that societal questions are absent from work on neurodivergent children.

Compared with the first corpus, which spans schools and society broadly, these three clusters are narrower and more clinically oriented. Their shared concern is equity: that children with autism, ADHD, and dyslexia have the tools, emotional support, and safeguards needed to take part fully in digital life.

When comparing the two analyses, we see a progression from general digital literacies to specialised, inclusive digital literacies. Research on children in general largely describes the general infrastructure of digital education, including teachers, parents, and the school system, whereas research on neurodivergent children focuses on the adaptations that allow children with particular cognitive profiles to use that same infrastructure.

### 3.3. Comparative Analysis: General vs. Neurodiversity Corpus (RQ2—RQ4)

The comparison was made at the level of themes, not of individual keywords, because themes are the output of SKS and are therefore the units on which the two corpora can be compared. We proceeded in three steps. First, we placed the themes of each corpus on the macro, meso and micro levels defined in Section 3.2.1, using the same criterion of scope of actors and setting. Second, we identified the dimensions on which both corpora carry content, namely scope, learning model, mode of communication, safety focus and primary actors, and recorded for each corpus what its themes and categories say on that dimension. These five dimensions are reported in Table 3. Third, we read the resulting contrasts and condensed them into the three shifts reported in Section 3.4. The comparison contrasts emphasise the two bodies of literature; it does not compare demonstrated effectiveness, and the difference in corpus size means that the absence of a topic from the NCC indicates limited research attention rather than an established gap in practice.

### 3.4. Three Shifts in Emphasis Between the Corpora (RQ2—RQ4)

The three shifts are the third step of the procedure in Section 3.3: they condense the contrasts in Table 3 into differences that hold across more than one dimension. The first difference concerns the locus of support. The general literature centres on instructional strategies, that is, on how teachers deliver a lesson, whereas the literature on neurodivergent children gives more weight to rapport building and digital parenting. This suggests that, for these children, the relationship and the emotional setting matter alongside the curriculum.

The second difference concerns online safety. The general literature promotes critical thinking so that children can identify misleading or hostile content, while research on neurodivergent children notes that reasoning about risk may not be sufficient where social-cognitive processing differs. It therefore emphasises protective mediations, that is, safeguards tailored to specific vulnerabilities, and links these to reported differences in executive control and social-emotional decoding [68,69].

The third difference concerns the conception of literacy itself. The general literature draws on applied linguistics to explain how children comprehend text, whereas research on neurodivergent children turns to implicit learning, for example among children with dyslexia. The emphasis moves from how best to teach a child to read toward how digital tools can support learning while reducing the frustration associated with conventional reading.

## 4. Discussion

Rather than restating these descriptive trends, this section considers what the contrast between the two corpora means. The disparity in volume is itself a first point of interpretation: children with autism, ADHD, and dyslexia remain comparatively under-served by a literature built around neurotypical learners. Because digital literacies increasingly shape children’s social participation, online safety, and emotional well-being, this gap is not only educational but also a matter of child-health equity, since the groups least served by universal models are often those most exposed to online risks such as cyberbullying and social isolation.

From a public health and paediatric prevention perspective, this disparity indicates that universal digital educational interventions are insufficient to support cohorts with divergent cognitive profiles. Within school medicine and clinical psychology, the necessity for structured, neurodiversity-affirming healthcare and guidance is increasingly emphasised [70]. A recurring pattern in this synthesis is a difference in emphasis between the two bodies of literature, rather than a demonstrated hierarchy of effectiveness. Whereas general literacy frameworks emphasise macro-level socio-educational goals, such as critical analysis of media, social inclusion, and formal, teacher-led instruction, the literature on neurodivergent children concentrates on a more specialised, clinically informed orientation. This body of work foregrounds the limits of rule-based instruction for learners who face distinct cognitive barriers and gives greater attention to relational mediation, in which rapport-building, specialised digital parenting, and gamified frameworks are presented as ways to support implicit learning. Future research could assess whether these approaches are more effective than conventional instruction.

Furthermore, while the general literature foregrounds critical thinking as the route to digital safety, the literature on neurodivergent children foregrounds targeted protective mediations, which are presented as ways to address vulnerabilities linked to impulsivity or differences in social-emotional decoding, factors that bear directly on these children’s online safety and mental health. Because children with ADHD or ASD show reported differences in executive control and emotional decoding [68,69], standard cognitive media strategies may be insufficient to address the resulting risks. Recent work in this area includes AI-assisted design of executive-function rehabilitation programmes for individuals with ADHD, an example of newer technology-supported developments rather than of established effectiveness [71]. This suggests that clinical teams, including paediatricians, school nurses, and child psychiatrists, may need to look beyond advice on screen-time limits toward guidance on affective digital parenting, which has been proposed as a way to reduce the risk of internet addiction and related psychosocial harm. Taken together, this suggests that supporting equitable and safe digital participation may require complementing universal literacy provision with inclusive pedagogies and affective, relational support, rather than treating either in isolation. For child-health practice, this points to a preventive role at the population level: paediatric, mental-health, and public-health services, working alongside schools and families, are well placed to identify neurodivergent children at heightened online risk and to build protective mediations into routine care and safeguarding.

## 5. Study Limitations

This study maps the research field rather than appraising individual studies, and several limitations apply.

- The search drew only on Scopus; although its coverage and metadata are strong, a single database may omit work indexed elsewhere, such as Web of Science, ERIC, or PsycINFO, and Scopus favours English-language outputs. The specialised corpus is also small, so the NCC and its clusters are exploratory rather than statistically generalisable, and because that search targeted autism, ADHD, and dyslexia, it may not reflect children with other neurodevelopmental profiles. The method adds a further constraint: SKS works from keywords, metadata, and abstracts, and so can miss nuances found only in full texts. In addition, children were operationalised only through the child* operator, without an age restriction, so the corpora may include work on wider youth populations. Finally, the set of NCC terms shown on the map was curated manually, and other analysts might have selected the terms differently. The analysis also relies on English-language metadata as indexed in Scopus, so terminology in records originally published in other languages may be rendered imprecisely, and this could not be checked against the original wording.
- Interpretation also carries limits. The labels we assign to the VOSviewer clusters depend on our reading of the dominant keywords and representative publications, and other analysts might group or name them differently, especially for the small NCC. More broadly, as a bibliometric mapping, the study shows where research attention is concentrated but cannot establish the comparative effectiveness of the pedagogical, parental, or safety approaches it identifies; statements about emphasis in the literature should therefore not be read as evidence of efficacy.
- The same applies to the macro, meso and micro ordering of the themes, which reflects our reading of their scope rather than a categorisation produced by the method.

## 6. Future Research

Several directions follow from this mapping. The most pressing is closer integration across disciplines: research on children’s digital literacies, clinical psychology, and special education still develops largely in parallel, and bringing these strands together would help translate descriptive patterns into coherent, age-appropriate curricula. Equally important is empirical testing. The protective mediations and digital-parenting strategies identified here have rarely been evaluated, and controlled studies are needed to establish whether, and for whom, they reduce cyberbullying and other online harms.

Longer-term questions also remain open. The effects of gamified and implicit-learning tools, such as digital storytelling, on both skill acquisition and socio-emotional resilience are best assessed through longitudinal designs rather than cross-sectional snapshots. Finally, current research is concentrated in the United States, the United Kingdom, and Australia, which limits what can be said about other settings; work in a wider range of regions would clarify how cultural context and resourcing shape digital inclusion for neurodivergent children.

## 7. Implications

The study has several practical implications for education and child health.

The first concerns pedagogy. For neurodivergent children, instruction designed around neurotypical learners is often a poor fit, and the literature points instead toward implicit learning and assistive approaches. Tools such as gamification and digital storytelling are presented in this literature as ways to help these children work around verbal or written barriers and express themselves through several modes at once, which suggests that inclusive teaching could treat such tools as part of the core repertoire rather than as optional extras.

Closely related is the question of safety. General digital-safety education relies on critical thinking to help children recognise online risks, but for neurodivergent children this may not be enough, since reported differences in social-emotional decoding and executive control (Section 4) can raise their exposure to risks such as cyberbullying. The literature therefore locates safety not in the curriculum alone but in the relational environment, and presents rapport-building and digital parenting as central to that support.

These two strands come together in educational design. The persistent double digital divide leaves children with special educational needs at the margins of digital life, and addressing it calls for an integrated public-health and socio-educational framework. Alongside universal provision through teachers, parents, and schools, institutions can embed inclusive pedagogy and targeted safety supports, balancing shared educational goals with the specific needs of neurodivergent children. For health services specifically, brief digital-resilience guidance could be added to paediatric and mental-health consultations, and clinicians could be supported to advise parents of neurodivergent children on protective mediation, so that these supports reach beyond the classroom into routine child-health practice.

Realising such a model will require work across disciplines. As noted in Section 6, the field is still emerging, so progress will depend on sustained collaboration among general education, clinical psychology, and special education to adapt mainstream literacy models to the needs of neurodivergent children.

## 8. Conclusions

This synthesis shows how thinking about children’s digital literacies has changed, moving away from the idea of an innate “digital native” competence toward a recognition that these skills need structured support. For children generally, the emphasis has shifted from acquiring functional skills to developing critical agency, and this depends on cooperation between schools and families that helps children move from passive use toward active, critical engagement with the media around them.

For neurodivergent children, including those with ASD, ADHD, or dyslexia, the picture is different. A persistent double digital divide leaves them poorly served by instruction built around neurotypical learners. Where the general field stresses teacher-led critical analysis, research on these children emphasises implicit learning and assistive approaches, with tools such as gamification and digital storytelling presented as ways to work around verbal or written barriers and to support expression in other modes.

Crucially, the literature on these vulnerable learners suggests that the focus of digital literacy extends beyond reasoning about risks to the provision of protective mediations. Because differences in social-emotional decoding or impulse control may heighten online risks, this work positions the emotional and relational environment (rapport-building and specialised digital parenting) as central to building resilience. The two corpora indicate the importance of an integrated public-health and socio-educational framework that complements universal socio-educational goals with inclusive pedagogy and targeted safety supports. Establishing how well such approaches protect children’s well-being is a task for future empirical work; what this synthesis offers is a map of where that work is most needed if the digital environment is to remain equitable, empowering, and safe for every child, regardless of neurocognitive profile.

## Figures and Tables

**Table 1:** Themes in the research of digital literacies–ACC.

Cluster Colour	Representative Keywords Related to Research Questions	Categories	Theme
Red	Digital literacy, Information literacy, Media literacy, Primary school, Parental mediation, Social media, Digital parenting, Digital inclusion, Digital competence, Cyberbullying, Media Education, Critical thinking, Children	Digital literacies and social media and digital divide and digital competence; Digital literacies and children; Digital literacies and parents; Child, digital literacies and COVID; Primary school and media and digital literacy; Parental mediation, digital competency and media and digital literacy; Digital parenting and digital literacy; Digital inclusion, digital divide and digital literacy; Cyberbullying, internet social media, and digital and media literacy; critical thinking and media education in children	The Socio-Educational Landscape of Digital, Information and Media Literacies
Green	Primary education, Childhood education, Family literacy, Technology, Digital media, Artificial intelligence, Digital literacies	Literacy and technology, Digital literacies, Learning and family literacy, Primary education and digital literacies, Digital literacies and artificial intelligence	Co-learning of digital literacies
Blue	Childhood, Teacher education, digital/media literacies, Critical literacy, Content literacy, Motivation/engagement, comprehension	New literacies and comprehension in childhood; digital/media literacies, teacher education, motivation/engagement, and comprehension and content literacy	Applied Linguistics and New Literacy Studies
Yellow	Childhood, new literacies–digital media, visual literacy–digital media, critical analysis–digital media	Digital media literacies and visual literacy in childhood; critical analysis–digital media in childhood	Digital empowerment in childhood
Violet	Instructional strategies, early childhood	Instructional strategies in early childhood	Applied Media Pedagogy

**Table 2:** Themes in the research of digital literacies–NCC.

Cluster Colour	Representative Keywords Related to Research Questions	Categories	Theme
Red and Yellow	Red: Dyslexia, Children, Implicit learning, Self-judgement of competence, Critical learning, Digital literacies, Digital age, Digital storytelling, Multilingual digital storytelling, Virtual reality, Special educational needs Yellow: Reading, Phonemic awareness, Phonological awareness, Orthographic transparency, Visual attention span, Dyslexia risk	Implicit learning and self-judgement of competence in dyslexic children Reading, phonemic and phonological awareness, orthographic transparency and visual attention span in relation to dyslexia risk Critical learning of digital literacies through digital storytelling, multilingual digital storytelling and virtual reality with children with special educational needs	Inclusive Digital Pedagogy and Implicit Language Acquisition in Neurodiverse Learners
Blue	Digital literacy, Gamification, Inclusive learning, Rapport building, Children with special needs, Phonological awareness	Gamification and inclusive learning in digital literacy for children with special needs Rapport building as a relational condition of digital literacy work	Relational and Gamified Frameworks for Inclusive Digital Literacy
Green	Cyberbullying, Internet safety, Learning disabilities, Autism spectrum, ADHD, Digital citizenship, Mental health, Ecological momentary intervention, Momentary symptom assessment	Cyberbullying and internet safety among children with learning disabilities, autism spectrum conditions and ADHD Digital citizenship as a protective mediation Mental health monitoring through ecological momentary intervention and momentary symptom assessment	Digital Risk and Protective Mediations for Neurodivergent Learners

**Table 3:** Comparative analysis of the general socio-educational corpus and the neurodiversity and inclusion corpus.

Feature	General Socio-Educational (Table 1)	Neurodiversity & Inclusion (Table 2)
Scope	Broad & Universal. Focuses on the general population of students and the “Digital Divide.”	Narrow & Clinical. Focuses on specific diagnoses like autism, ADHD, and dyslexia.
Learning Model	Formal & Social. Emphasises instruction, comprehension, and teacher-led education.	Implicit & Adaptive. Emphasises “unconscious” learning and gamification to bypass cognitive barriers.
Communication	Standard Literacies. Focuses on reading, writing, and critical thinking in social media.	Multimodal Expression. Focuses on “digital storytelling” as a substitute for traditional verbal/written limits.
Safety Focus	General Awareness. Deals with cyberbullying as a social issue for all children.	Targeted Vulnerability. Addresses how specific traits (e.g., ADHD impulsivity) increase online risks.
Primary Actors	Teachers, primary school students, and “the general parent.”	Special education experts, “digital parents” of SEN children, and the child as a unique learner.

## Data Availability

No new data were created or analyzed in this study. Data sharing is not applicable to this article.
